# Costs of living in metal polluted areas: respiration rate of the ground beetle *Pterostichus oblongopunctatus* from two gradients of metal pollution

**DOI:** 10.1007/s10646-012-1008-y

**Published:** 2012-10-23

**Authors:** Agnieszka J. Bednarska, Izabela Stachowicz

**Affiliations:** Institute of Environmental Sciences, Jagiellonian University, Gronostajowa 7, 30-387 Kraków, Poland

**Keywords:** Zinc, Cadmium, Carabids, Energetic costs

## Abstract

To address the question about costs of living in polluted areas, biomarkers linked to metabolism were measured in *Pterostichus oblongopunctatus* (Coleoptera: Carabidae) collected along two metal-pollution gradients in the vicinity of the two largest Polish zinc smelters: ‘Bolesław’ and ‘Miasteczko Śląskie’ in southern Poland. Both gradients covered a broad range of Zn and Cd concentrations in the humus layer (109–6151 and 1.48–71.4 mg kg^−1^, respectively) and body metal concentrations increased with increasing soil metal concentrations. The whole-organism respiration rate was measured as oxygen consumption with Micro-Oxymax respirometer, and cellular energy consumption—as the activity of electron transport system, which is linked to cellular respiration rate. The significant increase in the whole-organism respiration rate with the body metal concentration was found when taking into account other factors such as body mass, gradient (or year of sampling as the beetles were collected on the gradients in different years) and the interactions: body metal concentrations × collection date, body metal concentrations × body mass, and body mass × gradient/sampling year. However, no relationships between metal concentrations in soil or body metal concentrations and the whole-organism or cellular respiration rate could be detected when using mean values per site, underlining the crucial importance of incorporating individual variability in such analyses. The observed increase of the whole-organism respiration rate with increasing body contamination with metals suggests that *P. oblongopunctatus* incurs energetic expenditures resulting from the necessity to facilitate metal elimination or repair of toxicant-induced damage.

## Introduction

Metals are persistent pollutants, with strong direct and indirect impacts on invertebrates (Posthuma and van Straalen 1993). Direct physiological effects may occur by alteration or inhibition of various enzymatic pathways, while indirect effects can be not only physiological (e.g. lipid peroxidation caused by produced reactive oxygen species (Wilczek et al. 2004)) but may be also observed as pollution-driven changes in reproduction or survival due to diminished energy budget (Posthuma and Van Straalen 1993). Some indirect evidence of increased detoxification costs are available for invertebrates exposed to elevated metal concentrations, for example smaller body size of woodlice *Porcellio scaber* (Jones and Hopkin 1998) and lower reproduction of the ground beetle *Pterostichus oblongopunctatus* (Łagisz et al. 2002) or their decreased tolerance to other stressors (Stone et al. 2001).

Oxygen consumption is a direct measure of respiratory metabolism and allows, thus, for drawing conclusions about an organism’s maintenance costs (Calow 1991). Because the respiration rate is relatively easy to measure, it is often used as the equivalent for a metabolic rate (Migula 1989; Handy and Depledge 1999). It can serve as a convenient end-point in studies in which effect of toxicants (as well as other factors) on metabolic rate may be expected. A general prediction that results from models involving metabolically costly physiological responses is that metabolic rate should increase with increasing intoxication (exposure time and/or concentration) until irreversible pathological effects impair metabolism itself (Calow 1989). Indeed, laboratory studies on animals treated for several generations with cadmium (beet armyworm, *Spodoptera exigua*; Kramarz and Kafel 2003) or copper (confused flour beetle, *Tribolium confusum*; Lukasik and Laskowski 2007) revealed elevated whole-body respiration rates in the exposed individuals. Laskowski et al. (1996) showed that the respiration rate of centipedes treated with copper increased for only a short period and returned later to the same level as in control animals. In turn, Migula (1989) noticed a decrease in the respiratory metabolism in house crickets (*Acheta domesticus*) intoxicated with cadmium, while zinc and lead did not cause any effect.

Data on whole-body respiration rates of terrestrial invertebrates inhabiting metal polluted sites are scarce (Lagisz et al. 2005). Even less data are available for respiratory metabolism of terrestrial invertebrates measured at the cellular level. This can be done by measuring the activity of the electron transport system (ETS) in mitochondria. Although this technique was initially developed to estimate respiration of phytoplankton and zooplankton species (Kenner and Ahmed 1975) and then validated for *Daphnia magna* (De Coen and Janssen 1997), it can also be used for other invertebrates (Moolman et al. 2007; Olsen et al. 2007).

The aim of this study was to relate respiration rates measured at organismal and cellular levels in field-collected animals to metal pollution. The respiration rates were measured in the ground beetle *P. oblongopunctatus* (Coleoptera: Carabidae) collected from sites differently contaminated with metals. We hypothesized an increase in metabolic rates with increasing body metal contamination as a result of energetically costly processes of metal detoxification (Sibly and Calow 1989), such as, e.g., production of metallothioneins and metal-containing granules (Hopkin 1989; Walker et al. 2006). The respiration rates were measured at two different levels because they may indicate different effects. The effect on the whole-organism respiration rate (R) would represent the long-term effect of chronic exposure to the metals, while ETS—the instantaneous maximum respiration rate at the sampling time; we expected the ETS to be prone to rapid temporal changes in conditions—as is the case for most biochemical biomarkers.

## Materials and methods

### Sampling sites

To control, as far as possible, for stress factors other than metals, the study was performed on animals collected along two distinct transects traced from two metal smelters. The clear metal pollution gradient was found for both transects.

Adult beetles were collected from six sites along a metal-pollution gradient in the vicinity of the ‘Bolesław’ zinc smelter in Olkusz (OLK) area and from six sites around Miasteczko Śląskie (MSL) zinc smelter. The sampling sites were chosen based on Zn and Cd concentrations in the humus layer to provide a wide range of pollution levels (Stefanowicz et al. 2008; Tarasek 2011). The concentrations were (mg kg^−1^ dry weight): Zn, 109–6151 and Cd, 1.5–71.4 at OLK gradient and Zn, 155–2906 and Cd, 2.2–55 at MSL gradient (Table 1). Although the same unpolluted site was used in both gradients, it was labeled by different metal concentrations in the soil: metal concentrations measured by Stefanowicz et al. (2008) were used for the OLK unpolluted site, and metal concentrations measured by Tarasek (2011) were used for the MSL unpolluted site. All sites at both pollution gradients were located in Scots pine forests on sandy podsolized soils.

**Table 1:** Metal concentrations in the humus layer and in the ground beetles *P. oblongopuncatus* (mean ± SD) and calculated pollution indices (PI)^a^ along two metal pollution gradients

Gradient	Location	Distance from nearest smelter (km)	Metals in soil^b^			Metals in beetles			Whole-body respiration rate	Cellular respiration rate
			Cd	Zn	PI	Cd	Zn	BCI	(μl O_2_ h^−1^ g^−1^ bw)	
			(mg kg^−1^ dw)			(mg kg^−1^ dw)				
	50º17′N, 19º29′E	1.9	71.4	6,151	52.3	2.5 ± 2.21 (23)^c^	115 ± 15 (24)	2.25	449 ± 74 (24)	4,771 ± 2,121
	50º18′N, 19º29′E	3.9	39.1	1,763	21.3	1.6 ± 1.11 (24)	107 ± 15 (23)	1.61	447 ± 55 (24)	5,282 ± 1,929
OLK	50º19′N, 19º30′E	5.3	14.7	1,253	10.7	1.6 ± 0.97 (24)	103 ± 10 (23)	1.62	442 ± 54 (23)	4,106 ± 2,262
	50º19′N, 19º32′E	7.9	12.2	755	7.6	1.8 ± 1.10 (24)	104 ± 15 (24)	1.73	423 ± 54 (23)	6,019 ± 2,792
	50º25′N, 19º38′E	19.6	4.03	224	2.4	0.6 ± 0.48 (23)	99 ± 90 (24)	0.94	452 ± 59 (24)	5,026 ± 2,112
Unpolluted site 2008	50º32′N, 19º39′E	31.8	1.48	109	1.0	0.7 ± 0.65 (23)	100 ± 11 (24)	1.00	432 ± 59 (24)	4,995 ± 2,046
	50º29′N, 18º57′E	2.1	52 ± 10.6	2,684 ± 689	20.5	3.7 ± 1.89 (32)	104 ± 16 (32)	4.76	535 ± 97 (30)	4,453 ± 2,855
	50º31′N, 18º56′E	2.6	55 ± 12.1	2,906 ± 726	21.9	3.4 ± 1.41 (38)	106 ± 17 (42)	4.39	562 ± 127 (40)	6,092 ± 3,164
MSL	50º31′N, 18º57′E	3.3	36 ± 10.1	1,886 ± 520	14.3	2.3 ± 1.19 (51)	101 ± 15 (54)	3.12	506 ± 89 (52)	3,566 ± 1,829
	50º32′N, 18º57′E	5.1	5,9 ± 1.5	292 ± 68	2.3	1.4 ± 0.51 (23)	105 ± 16 (24)	2.12	511 ± 78 (21)	3,880 ± 2,059
	50º34′N, 19º58′E	8.7	4.8 ± 2.3	319 ± 80	2.1	0.9 ± 0.44 (35)	100 ± 15 (36)	1.53	525 ± 118 (33)	3,165 ± 1,776
Unpolluted site 2010	50º32′N, 19º39′E	31.8	2.2 ± 1.8	155 ± 28	1.0	0.4 ± 0.37 (41)	95 ± 14 (43)	1.00	512 ± 110 (40)	4,536 ± 1,626

*OLK* Olkusz, *MSL* Miasteczko Slaskie

^a^Pollution and Body Concentration indices were calculated according to the equation ${\text{PI}} = \frac{{{\text{Zn}}_{i} /{\text{Zn}}_{U} + {\text{Cd}}_{i} /{\text{Cd}}_{U} }}{2}$, where index *i* denotes site number, index *U* unpolluted site, and metal symbols stand for their concentrations in soil (PI) or beetles (BCI) (mg kg^−1^ dry wt)

^b^Data for soil concentration for OLK gradient from Stefanowicz et al. (2008) and for MSL gradient from Tarasek (2011), no SD indicated for OLK gradient as chemical analysis were performed on mixed samples

^c^Number of beetles analyzed after outlier elimination

The beetles were sampled with pitfall traps. Ninety traps were distributed per site and emptied every second or third day between April 24 and May 15, 2008 at the OLK transect, and in the same period in 2010 at the MSL transect. After transporting to the laboratory, the beetles were separated by sex, placed individually in 30-ml plastic vials and kept in a controlled temperature chamber (20 °C, 60 % relative humidity, 16:8 light:dark photoperiod) for 24 h to void gut contents. The beetles were then weighed to the nearest 0.0001 g on an electronic balance (AS 160/C/2 Radwag, Poland). Because of the limited number of chambers in the respirometer (30), R was measured in a maximum of five males from each site and sampling date. After measurements, the beetles were weighed and frozen at −20 °C for metal analysis. The body concentrations of Zn and Cd were analyzed by flame (Zn) or by graphite furnace (Cd) atomic absorption spectrometry (Perkin-Elmer AAnalyst 800) after wet digestion in boiling HNO_3_, as described by Bednarska et al. (2012). The beetles used for ETS analysis (ten males per site) were frozen in liquid nitrogen after 24-h starvation and stored at −80 °C.

### Respiration rate measurements

The beetles were placed individually into 50 ml flasks connected to a 30-channel computer-controlled, closed-circuit Micro-Oxymax respirometer (Columbus Instruments, USA). The animals from different sites were assigned to the flasks at random. Along with the beetles, a punctured Eppendorf tube filled with distilled water and a hole in the lid was placed in each bottle to prevent desiccation. The respiration rate was measured over 28 h at 4-h intervals at 16:8 L:D and 20 °C. Respiration rate was measured as oxygen consumption per hour per beetle and then recalculated per gram body mass for data analysis (μl O_2_ g^−1^ h^−1^). The data were not corrected for oxygen consumption by microbial growth in the flasks; our previous study indicated that the oxygen consumption in a control flask (without beetles but with Eppendorf-type tube with distilled water) was below 10 % of the flask with the beetle. Prior to data analysis, the first measurement point (the first 4-h interval) for each individual was discarded, since we suspected that the change of the environment and handling stress might temporarily cause abnormal activity and respiration rates. Animals that died in the course of the measurements were excluded from the study.

### Cellular respiration rate measurements

The energy consumption or cellular respiration rate was determined by measuring the activity of ETS. The ETS consists of a complex chain of macroenzymes (cytochromes, flavoproteins) in cell mitochondria that transport electrons for energy production. Since the synthesis and degradation of these macroenzymes is a function of the respiratory requirements of the organism, measuring ETS activity (also known as dehydrogenase activity) provides a time-averaged value of the maximum oxygen uptake rate (De Coen and Janssen 2003). The ETS was measured according to the method developed by De Coen and Janssen (1997) with minor modifications as described below.

Legs, elytra, and wings were carefully removed from each beetle using forceps and a scalpel and the remaining body parts of each individual were homogenized on ice using a PRO 200 mechanical homogenizer (Bioeko, Poland). The samples were homogenized on ice in 600 μl of ice-cold homogenizing buffer [0.08 M Tris–HCL pH 8.5, 15 % (w/v) Poly Vinyl Pyrrolidone, 153 μM MgSO_4_, and 0.2 % (w/v) Triton X-100]. After centrifugation (1,000×*g*., 10 min, 4 °C), 50 μl of diluted supernatant was added to 150 μl buffered substrate solution [0.13 mM Tris–HCl, 0.3 % (w/v) Triton X-100, pH 8.5, 1.7 mM NADH and 250 μM NADPH]. The colorimetric reaction was started by adding 100 μl of reagent solution [8 mM 2-p-iodo-phenyl-3-p-nitrophenyl 5-phenyl tetrazolium chloride, INT] and the absorbance was measured kinetically at 490 nm every 36 s for 3 min at 20 °C. The formazan production was determined from absorbance of the sample against the blank by using ε = 15,900 M^−1^ cm^−1^.

The cellular respiration rate was determined from ETS data, based on the theoretical stoichiometric relationship that for each 2 μM of formazan formed, 1 μM of oxygen is consumed by the electron transport system. The quantity of oxygen consumed was expressed per g body mass (μl O_2_ g^−1^ h^−1^).

### Statistical analysis

Prior to statistical analysis, outliers with the absolute values of modified MAD z-score greater than 3.5 were excluded and the distributions of the remaining data were checked for normality with Shapiro–Wilk’s *W* test. When this condition was not met, statistical analyses were performed on log-transformed data.

Correlations between Zn and Cd concentrations in soil and in the beetles were checked using Pearson correlation analysis. Because the metals in the soil were highly correlated with each other (*r* = 0.99, *p* < 0.0001) and with body concentrations (*r* in the range 0.76–0.91, *p* ≤ 0.003), the study sites were described with a single measure of pollution by defining a Pollution Index (PI):

$$ {\text{PI}} = \frac{{{\text{Zn}}_{i} /{\text{Zn}}_{U} + {\text{Cd}}_{i} /{\text{Cd}}_{U} }}{2} $$

where index *i* denotes site number at the particular transect, index_* U*_ site with the lowest metal concentration at this transect (unpolluted site), and metal symbols stand for their concentrations in soil. Different metal concentrations for the same unpolluted site in 2008 and 2010 were used to take into account possible temporal effects and differences in soil sampling and metal analysis procedures done by different people.

Similarly, a high correlation between Zn and Cd body concentrations (*r* = 0.8, *p* = 0.004) was found and a body contamination index (BCI) was calculated to summarize internal concentrations of metals in the beetles originating from each study site:

$$ {\text{BCI}} = \frac{{{\text{Zn}}_{i} /{\text{Zn}}_{U} + {\text{Cd}}_{i} /{\text{Cd}}_{U} }}{2} $$

where metal symbols stand for their concentrations in beetles. In addition, BCI_*i*_ was calculated for each individual separately in a similar way, but Zn_*i*_ and Cd_*i*_ denoted internal concentrations of metals in individual *i*, and instead of site with the lowest metal concentration at the gradient (unpolluted site)—the beetle with the lowest concentration at the transect was used (Zn_min_ and Cd_min_).

The simple regression analysis was performed to verify the effect of pollution (expressed as PI) on body metal concentrations (expressed as BCI) across all studied sites. The comparison of the regression lines was performed to check for possible effect of the sampling at two different gradients/years on the impact of metal pollution on metal accumulation.

Because the beetles were collected from the same unpolluted site in 2008 and 2010, possible differences in respiration rates between the years which might result from factors other than metal pollution, were tested for this site using a *t* test.

Multiple-regression was used to find out which variables and their interactions affected the endpoints measured. The independent variables in the model for R were: BCI_*i*_, body mass, collection day and gradient (or sampling year, as it was not possible to separate the effect of different years of sampling from the gradient as such, due to the beetle sampling schedule: the beetles were collected on each gradient in different years). The interactions and variables with the highest p value were removed consecutively from the model (backward stepwise procedure with cut-off value *F* = 4.0) as long there were any interactions/variables with *p* > 0.05.

Due to technical reasons the concentrations of Cd and Zn could not be measured in the same animals in which ETS values were assessed. Therefore, the multiple regression analysis for ETS was done on mean values with PI and gradient as independent variables. Because of this limitation, neither body mass nor collection day could be included in the analysis. For comparison, also R was re-analyzed in that way.

The relationship between mean R and mean ETS was checked using reduced major axis regression (RMA).

All statistical analyses except RMA were performed with Statgraphics Centurion XVI (StatPoint Technologies, Inc., USA), and PAST (http://folk.uio.no/ohammer/past) was used for RMA.

## Results

Among 144 values of body Zn or Cd concentrations at the OLK gradient, three values for Cd and two for Zn were excluded from statistical analysis as outliers. For MSL (*n* = 231), eleven outliers for body Cd concentration were excluded. In respiration rate measurements seven beetles (one from OLK and six from MSL) which died and ten outliers (one from OLK and nine from MSL) were excluded from statistical analysis.

The highest concentrations of Zn and Cd in soil were found at OLK gradient at the site nearest to the smelter (6,151 and 71.4 mg kg^−1^, respectively). The highest body concentrations of Zn were found in beetles collected from the Olkusz gradient (mean 115 mg kg^−1^ dry bw), whereas beetles collected near Miasteczko Śląskie smelter accumulated the highest concentrations of Cd (mean 3.7 mg kg^−1^ dry bw). Detailed data on the metal concentrations in the soils and beetles are given in Table 1.

**Fig. 1:**
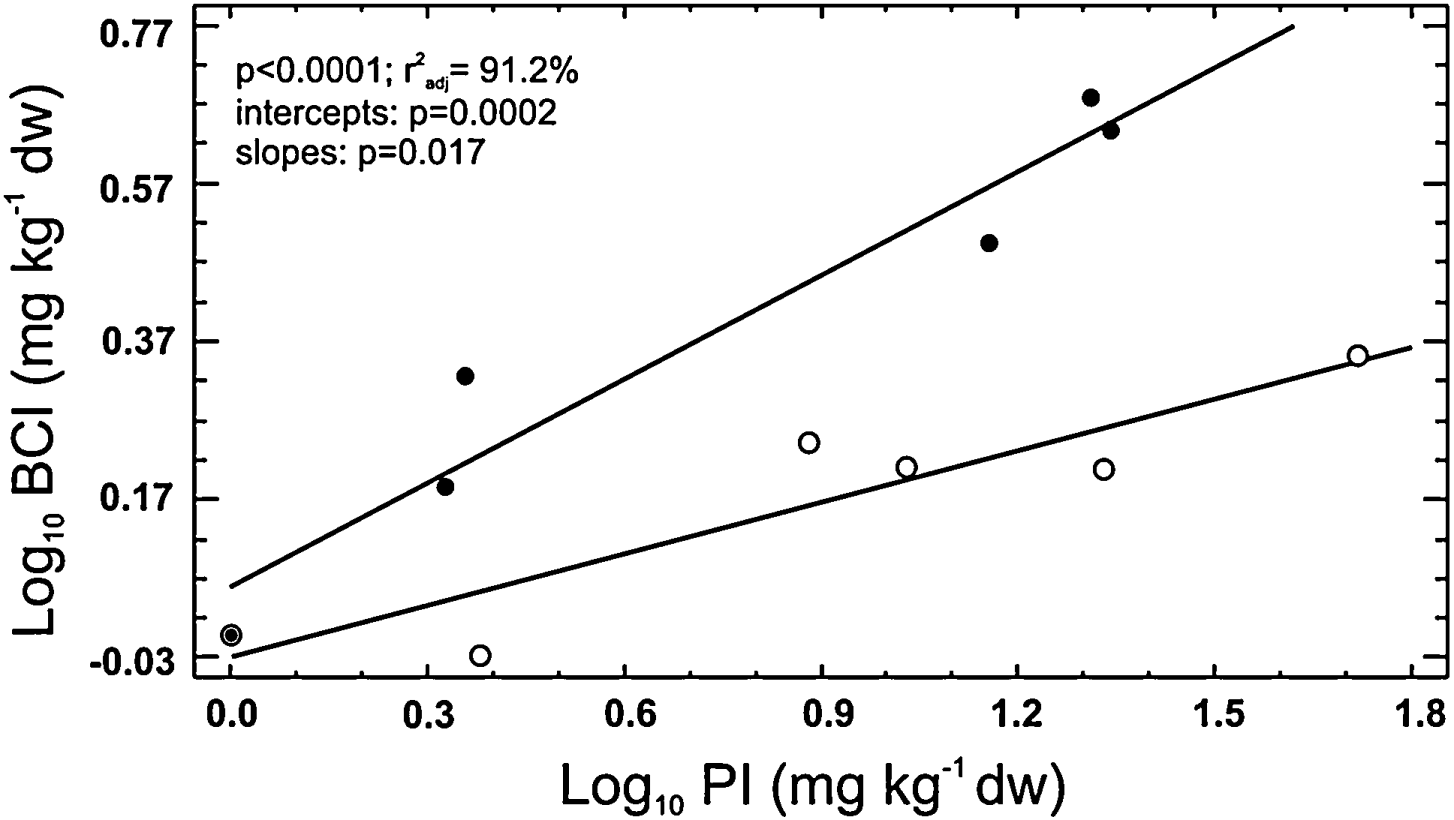
Effect of metal concentration in soil (expressed as pollution index, PI) on the internal body concentration of metals (expressed as body concentration index, BCI) in *P. oblongopunctatus* collected along two pollution gradients (*p* < 0.0001, ${\text{r}}_{adj}^{2}$ = 91.2 %, difference in intercepts: *p* = 0.0002, difference in slopes: *p* = 0.017); *open circles*, OLK; *full circles*, MSL

The BCI increased with increasing PI (*p* = 0.009, *r* ^2^ = 51.5 %) and significant differences between the gradients were found in both regression intercepts (*p* = 0.0002) and slopes (*p* = 0.017). The beetles at MSL gradient had generally higher BCI and its increase was steeper (Fig. 1). The model explained 93.6 % of total variance (${\text{r}}_{adj}^{2}$ = 91.2 %) and was highly significant (*p* < 0.0001).

A significant difference was found in R between the two sampling years at the unpolluted site: the beetles collected in 2010 had higher respiration rate than those collected in 2008 (*p* = 0.01). The cellular respiration rates exhibited large variation (Table 1) and no difference between the years was found.

**Fig. 2:**
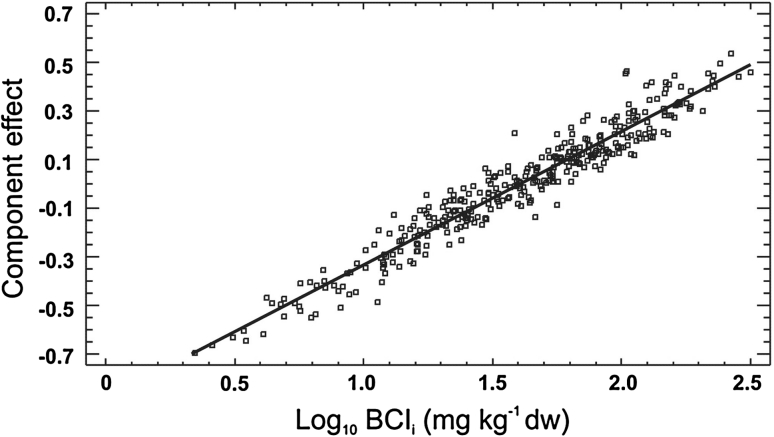
Results of the multiple regression analysis: effect of body concentration of metals expressed as body concentration index, BCI_*i*_ on the whole-body respiration rate of *P. oblongopunctatus* collected along two pollution gradients. The *line* shows the relative change in the predicted values of respiration rates that occurs when changing body concentration of metals over their observed ranges. *Each point* is then plotted by adding its residuals to a line. The respiration rate correlates with BCI_*i*_ at *p* = 0.014. The model included also body mass (*p* < 0.0001), year (or gradient) (*p* = 0.0025) and interactions: BCI_*i*_ × wet body mass (*p* = 0.018), BCI_*i*_ × collection day (*p* = 0.006) and year (or gradient) × wet body mass (*p* = 0.014), and explained 38.7 % of the total variability (${\text{r}}_{adj}^{2}$ = 37.6 %)

R increased with increasing BCI_*i*_ (*p* = 0.014, Fig. 2). The model included also body mass (*p* < 0.0001), year/gradient (*p* = 0.0025) and interactions: BCI_*i*_ × wet body mass (*p* = 0.018), BCI_*i*_ × collection day (*p* = 0.006) and gradient (or sampling year) × wet body mass (*p* = 0.014) (Table 2), and explained 38.7 % of the total variability (${\text{r}}_{adj}^{2}$ = 37.6 %).

**Table 2:** The results of fitting a multiple linear regression model to describe the relationship between the whole-body respiration rate and independent variables and their interactions

Model term	Coefficient estimates	SE	Statistic *T*	*p* value
Constant	0.796	0.3568	2.229	0.026
BCI_*i*_	0.550	0.2228	2.467	0.014
Body mass	−1.400	0.2665	−5.252	<0.0001
Year/gradient	0.350	0.1146	3.052	0.0025
BCI_*i*_ × wet body mass	0.511	0.1622	3.149	0.018
BCI_*i*_ × collection day	0.001	0.0004	2.791	0.006
Year/gradient × wet body mass	0.278	0.086	3.217	0.014

Neither PI nor gradient/sampling year was found to affect mean ETS, but the mean R was lower at OLK (2008 sampling) than at MSL gradient (2010 sampling) (*p* < 0.0001, ${\text{r}}_{adj}^{2}$ = 88.2 %). No significant relationship between mean R and mean ETS was found.

## Discussion

Chronic exposure to environmental pollutants doesn’t have to be lethal and some species of carabids are able to maintain viable populations in areas highly contaminated with metals (Skalski et al. 2011). However, they may still incur physiological costs associated with metal exposure. There are a priori grounds for expecting physiological costs associated with processes deployed to cope with exposure to contaminants (Forbes and Calow 1996). It is generally accepted that coping with stress (defense) or repairing damages (alterations) implies energy costs connected with re-allocation of resources favoring tolerance to stress (Calow 1991). The energy expended to defense is no longer available for maintenance, growth and reproduction, suggesting potential effects on the health status of organisms and the fate of populations. Indeed, populations of *P. oblongopunctatus* inhabiting metal-polluted areas on the OLK gradient have been previously demonstrated to be sensitive to the additional stressors (Stone et al. 2001) and have reduced fertility (Łagisz and Laskowski 2002).

The depletion of an animal’s energy reserves or increase in its metabolic rates have been associated with the toxicity of metals, such as cadmium and zinc (Moolman et al. 2007). In our previous study we did not observe the depletion of energy reserves with increasing metal contamination in the beetles collected along the same gradients of pollution (Bednarska et al. 2012). This study, however, has demonstrated significant energetic costs associated with living in metal contaminated areas. Given that respiration rate is an indirect measure of an organism’s maintenance costs, the observed increase of R with increasing BCI_*i*_ suggests that *P. oblongopunctatus* incurred energetic expenditures, presumably as a result of energy required to facilitate metal elimination or repair toxicant-induced cellular damage (Calow 1991). The metabolic (and fitness) costs associated with metal resistance were also observed in other species (harbour ragworms *Nereis diversicolor*, Pook et al. 2009; bullfrog tadpoles *Rana catesbeiana*, Rowe et al. 1998). The increase of R with increasing BCI_*i*_, but lack of such relationships between metal concentrations in soil or body metal concentrations and the whole-organism or cellular respiration rate when using mean values per site, suggest the importance of incorporating individual variability in analyses of respiration rate data.

Although the liner relationship between R and ETS was shown for 15 marine zooplankton species (King and Packard 1975), we did not find such a relationship in *P. oblongopunctatus*. In general, we found much higher ETS values compared to R. This can be explained by the fact that the applied method measures the maximum ETS activity under saturated substrate concentrations. Thus, ETS is a biochemical measure of the potential maximal metabolic activity and it shows the value of oxygen consumption that would occur if all enzymes worked at their maximum capacity. The energy consumed at the cellular level is measured as instantaneous enzyme activity which is a snapshot view of energy consumption but not integration over time of what the cell has actually consumed (Olsen et al. 2007). On the other hand, R can represent long-term effects because organisms have to handle toxic chemicals as long as their body concentrations are elevated and even after their detoxification and/or excretion, damage repair may still require significant amounts of energy (Forbes and Calow 1996).

Respiratory metabolism is one of the main components of an energy budget and, at the same time, one of the most sensitive to both internal and external factors (e.g., sex, body mass, physiological state) (Chaabane et al. 1999). Therefore, to avoid possible large variation caused by sex-specific metabolism and physiological state of beetles, only males were used in this study. The body mass of beetles and collection date were taken into account in statistical analysis of R. However, it was not possible to collect and measure animals from both gradients in the same year, so we could not avoid the possible differences between the years, e.g., in weather conditions. Indeed, the higher whole-body respiration rate in control beetles collected in 2010 could be, at least partly, the consequence of a severe winter preceding the 2010 sampling (meteorological data from the Research Station of the Institute of Geography and Spatial Management, Jagiellonian University, in Gaik-Brzezowa, Wieliczka Foothills). Our earlier laboratory study on the same species showed that the temperature at which the beetles were cultured affected their respiration, even if the respiration rate was always measured at the same constant temperature for all beetles (20 °C): animals originating from lower temperatures had higher respiration rates (Bednarska and Laskowski 2008). It is not possible to discriminate between the effect of year (e.g., difference in the weather) and the effect of gradient as such in the present study. However, irrespectively of possible differences between the years or gradients, the strong relationship between R and BCI_*i*_ found in this study allows for more general conclusions about the cost, in bioenergetic terms, of tolerance to metals. In addition, our findings are consistent with physiological responses documented by Łagisz and Laskowski (2002) for the same species. The authors found a positive relationship between respiration rates of males of *P. oblongopunctatus* from an F1 generation fed uncontaminated food in the laboratory and zinc concentration at the sites from which their parents were collected. Although the study by Łagisz and Laskowski (2002) attempted to separate the effect of possible adaptation to metal contaminated environment from the direct toxic effect of metals, their further study did not reveal the genetic adaptation to metal pollution (Lagisz and Laskowski 2008).

To conclude, our results suggest that *P. oblongopunctatus* are able to survive in metal-polluted environments, but coping with high body metal concentrations has significant impact on their energy metabolism.
